# Did the New Italian Law on Mandatory Vaccines Affect Adverse Event Following Immunization’s Reporting? A Pharmacovigilance Study in Southern Italy

**DOI:** 10.3389/fphar.2018.01003

**Published:** 2018-09-04

**Authors:** Cristina Scavone, Concetta Rafaniello, Simona Brusco, Michele Bertini, Enrica Menditto, Valentina Orlando, Ugo Trama, Liberata Sportiello, Francesco Rossi, Annalisa Capuano

**Affiliations:** ^1^Department of Experimental Medicine, Section of Pharmacology “L. Donatelli,” Campania Regional Center for Pharmacovigilance and Pharmacoepidemiology, University of Campania “Luigi Vanvitelli”, Naples, Italy; ^2^CIRFF, Center of Pharmacoeconomics, University of Naples Federico II, Naples, Italy; ^3^Regional Pharmaceutical Unit, Campania Region, Naples, Italy

**Keywords:** mandatory vaccines, safety, Italian pharmacovigilance, RNF, AEFI

## Abstract

Despite the well-recognized role of vaccines, coverage is far from optimal especially in children, representing a growing concern also in Italy. In order to reverse this emergency, the Italian Ministry approved in July 2017 the Law 119/2017, which renders mandatory and free of charge 10 vaccinations for patients aged 0–16. We aim to investigate the effects of the new Law 119/2017 on the reporting of adverse events following immunization related to mandatory vaccines into the Italian Pharmacovigilance database (Rete Nazionale di Farmacovigilanza – RNF). Therefore, we analyzed the spontaneous reports of suspected adverse events following immunization recorded in Campania Region (South of Italy) from December 1, 2016, to March 31, 2018. During the study period, 69 reports, covering 179 AEFIs, related to mandatory vaccines were sent to Campania Pharmacovigilance Regional Center. A substantial increase in AEFIs reporting was observed after the adoption of Law 119/2017. Out of 69 reports, 62% reported AEFIs that were considered as not serious and 78% had a favorable outcome. Out of 179 AEFIs, more than half referred to the following SOC: “general disorders and administration site conditions,” “nervous system disorders,” and “psychiatric disorders.” The highest number of reports came from patient/citizen. After the adoption of the Law 119/2017, there was an increase in the number of reports (18 before the adoption of the Law vs. 51 after). According to reported AEFIs during the entire period, no worrying safety data have emerged. In our opinion, the increase in the number of AEFIs’ reports should be related to the increase in vaccination coverage as well as to the intense debate that has followed the new Law. In this context, the continuous monitoring of vaccine safety and the fully implementation of vaccine–vigilance programs play a key role in achieving higher confidence in immunization programs and optimal vaccination coverage rate.

## Introduction

It is worldwide recognized that vaccinations have represented one of the most important breakthrough for global health, leading to significant reductions in mortality and morbidity in the general population (Parretta et al., 2011; Pellegrino et al., 2015; World Health Organization, 2018). Despite their well-recognized role, coverage for highly recommended vaccines is far from optimal especially in children (Greenwood, 2014; World Health Organization, 2017), representing a growing concern also in Italy. Data shared by the Italian Ministry of Health revealed that, during 2017, 4,885 measles cases and 4 deaths were observed; among those cases, 88% were unvaccinated and 6% were vaccinated with only one dose, contrary to what recommended by the National Vaccination Prevention Plan (PNPV) 2017–2019 (Ministero Della Salute, 2017c; Piano Nazionale Prevenzione Vaccinale, 2017). Except for pneumococcal and meningococcal coverage, the negative trend of vaccination in Italy involved both mandatory vaccinations (diphtheria, polio, tetanus, hepatitis B), and some of those recommended (Pezzotti et al., 2018). In Campania Region (a region in the South of Italy, which accounts for more than 5 million inhabitants and includes five provinces) the coverage for vaccinations scheduled within 36 months of age (update June 2017) was 92.72% for poliomyelitis, 92.71% for diphtheria, tetanus, pertussis, and hepatitis B, 92.54% for *H. influenzae* B, 84.01% for measles, mumps and rubella (MMR), 44.5% for varicella (within 24 months of age) (Epicentro, 2018). The reason of reduction in vaccination coverage is to be found in the almost complete disappearance of several preventable diseases that has reduced worldwide the perception of the danger of contagion and have simultaneously facilitated the spread of opposition movements to vaccinations, mainly for ethical reasons and for fear of adverse events following immunization (AEFIs). According to World Health Organization (WHO), the latter are defined as “any untoward medical occurrence which follows immunization and which does not necessarily have a causal relationship with the usage of the vaccine” (World Health Organization, 2012).

In order to reverse this emergency, the Italian Ministry approved on July 31, 2017, a new law (Law 119/2017 – GU Serie Generale n.182 del August 5, 2017), which renders mandatory and free of charge 10 vaccinations for all patients aged 0–16 (diphtheria, polio, tetanus, hepatitis B, pertussis, *H. influenzae* B, measles, mumps, rubella, and varicella). The obligation for MMR, and varicella vaccines will be reviewed every three years based on epidemiological data and reached vaccination coverage. Additionally, further vaccinations are strongly recommended in specific age groups of patients (Ministero Della Salute, 2017a,b). The new Law brings novelties also for school registration and attendance. On March 2018, the deadline for the fulfillment of compulsory vaccinations for kindergarten attendance expired. After the fully implementation of this Law, the National Institute of Health (Istituto Superiore di Sanità) shared new data on vaccination coverage revealing that in 2018 the goal of the Law 119/2017 has been achieved in 11 out of 21 Italian Regions, including Campania. Although the new Law allowed achieving higher vaccination coverage, it has attracted criticism both from the anti-vaccine groups and politicians due to its imperative modalities (Scavone et al., 2018). Considering that the rate of ADRs’ reports may increase in response to media attention and increased public awareness (CDC, 2018), we aim to investigate the effects of the new Law 119/2017 on the reporting of AEFIs related to mandatory vaccines into the Italian National Pharmacovigilance Network (Rete Nazionale di Farmacovigilanza, RNF), coordinated by the Italian Medicines Agency (AIFA). In order to improve pharmacovigilance activities in Italy, AIFA has established Pharmacovigilance Regional Centers (Mazzitello et al., 2013). Such structures are fully involved in the evaluation of ADRs/AEFIs’ reports coming from each region, in terms of quality of data, evaluation of causality assessment for each drug or vaccine/event couple, and contribution to signal analysis on drugs and vaccines. For the vaccine causality assessment, Pharmacovigilance Regional Centers use a standardized algorithm updated by the Global Advisory Committee on Vaccine Safety in 2013. According to this algorithm, the relation between vaccine and AEFI could be categorized as “consistent causal association to immunization,” “indeterminate,” “inconsistent causal association to immunization,” and “not classifiable” (WHO, 2013; Sessa et al., 2016a,b).

For this study, we analyzed the spontaneous reports of suspected AEFIs recorded in Campania Pharmacovigilance Regional Center from December 1, 2016, to March 31, 2018.

## Materials and Methods

### Data Source

Regional safety data were obtained from the RNF, which was established by the AIFA in 2001. Physicians, other healthcare professionals and patients/citizens, through a standardized reporting form, can send reports of suspected ADR and AEFI. This form contains details of the subject who experienced the adverse event (age, sex, medical history, etc.), the suspected ADR(s)/AEFI(s) (description of signs and symptoms or diagnosis, seriousness, outcome, etc.), the suspected drug(s)/vaccine(s), or any concomitant drug/vaccine as well as previous or current patient medical conditions.

### Descriptive Analysis and Case-Series

All AEFIs’ reports which reported mandatory vaccines (*H. influenzae* type B, measles, mumps, rubella, varicella, pertussis, diphtheria, tetanus, polio, and hepatitis B) as suspected and validated by the Campania Pharmacovigilance Regional Center from December 1, 2016, to March 31, 2018, were selected. We performed a descriptive analysis of those reports, stratifying by month/year, suspected vaccine, median age, gender, seriousness (serious – death; serious – hospitalization or its prolongation; serious – severe or permanent disability; serious – life-threat; serious – congenital abnormalities/birth deficits; serious – clinically relevant; not serious), outcome (favorable: completely resolved or improved; unfavorable: resolved with sequelae or unchanged), system organ class (SOC), preferred term (PT), source of report, and causality assessment. The choice of reference period was made in order to compare the main features of AEFIs’ reports entry into RNF during the 8 months before the approval of the Law 119/2017 (period 1: December 2016 to July 2017) vs. AEFIs’ reports entry during the 8 months after the approval of the Law 119/2017 (period 2: August 2017 to March 2018).

The sum of the number of reports for each vaccine may exceed the total number of reports found in the RNF since more than one vaccine can be reported as suspected in one single report. And equally, the sum of the number of AEFIs and SOCs may exceed the total number of reports since more than one AEFI and SOC can be reported in one single reports. Finally, since in each report seriousness is reported only once (independently by the number of AEFIs), it will be normal to find a number of seriousness degree equal to the number of reports and inferior to the number of AEFIs.

### Data Analysis

A descriptive analysis of all AEFIs reported during the study period 1 (from December 2016 to July 2017) and period 2 (from August 2017 to March 2018) was performed. Chi-squared analysis with Yates’ continuity correction or Fisher’s exact test, where appropriate, was employed to examine differences in the rate of AEFI’s report according to the two periods. A 5% significance level was considered for analysis. Data were analyzed using the software SPSS version 21.

### Compliance with Ethical Standards

Safety data deriving from the Italian spontaneous reporting system are anonymous and compliant with the ethical standard. Therefore, no further ethical measures were required.

## Results

**FIGURE 1:**
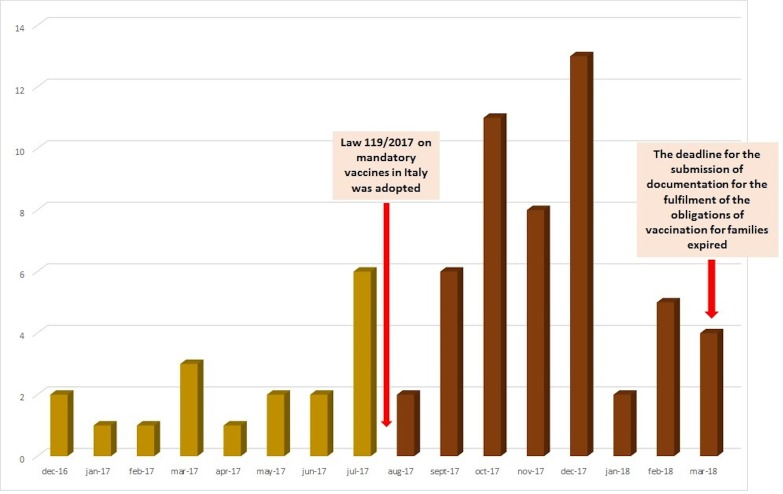
Trend of reports for mandatory vaccines in Campania Region (December 2016–March 2018).

From December 1, 2016, to March 31, 2018, 69 reports, covering 179 AEFIs, related to mandatory vaccines were sent to Campania Pharmacovigilance Regional Center. A substantial increase in AEFIs reporting was observed after the adoption of the Law 119/2017 (18 reports during period 1 vs. 51 reports during period 2) (**Figure 1** and **Table 1**).

**Table 1:** Main features of reports related to mandatory vaccine in Campania region.

	Total AEFIs’ Reports	AEFIs’ Reports During Period 1	AEFIs’ Reports During Period 2	*p*-Value
	**N. 69 (100)**	**N. 18 (26)**	**N. 51 (74)**	0.01
Median age (years)	1	1 (IQR 0.80–5.00)	1 (IQR 0.42–5.00)	0.459^$^
Female gender (%)	46	39	49	0.585^#^
**Seriousness of AEFIs’ reports**	**N. 65^∗^ (100)**	**N. 15 (23)**	**N. 50 (77)**	
Not serious	40 (62)	8 (53)	32 (64)	0.456
Serious	25 (38)	7 (47)	18 (36)	
**Outcome of AEFIs’ reports**	**N. 55^∗^ (100)**	**N. 14 (25)**	**N. 41 (75)**	
Favorable outcome^a^	43 (78)	11 (79)	32 (78)	0.967
Unfavorable outcome^a^	12 (22)	3 (21)	9 (22)	
**Source of AEFIs’ reports**	**N. 67^∗^ (100)**	**N. 18 (27)**	**N. 49 (73)**	
Healthcare professional	40 (60)	12 (67)	28 (57)	0.481
Patient/citizen	27 (40)	6 (33)	21 (43)	

*^∗^Total number of AEFIs’ reports for which data on seriousness, outcome, and source were reported. ^$^Mann–Whitney U test. ^#^Fisher exact test. Yates’ correction and Fisher exact test were used for the analyses. ^a^Favorable outcome: completely resolved/improved; unfavorable outcome: resolved with sequelae/unchanged.*

Overall, the median age of patients who experienced AEFIs was 1 year, and 46% were female. No differences in median age and gender between reports sent to the RNF during period 1 and 2 were found (**Table 1**). All considered suspected vaccines (mandatory ones) are reported in **Table 2**. The most commonly suspected ones were diphtheria, tetanus, pertussis, hepatitis B, poliomyelitis, and *H. influenzae* B vaccine and MMR vaccine (24 reports each one), followed by diphtheria, tetanus, and pertussis vaccine (12 reports), and diphtheria, tetanus, pertussis, and inactivated poliomyelitis vaccine (8 reports) (**Table 2**). As reported in **Table 2**, no differences were found in the number of AEFIs’ reports associated to individual suspected vaccine between the period 1 and 2. Out of 65 reports for which seriousness degree was reported, 62% reported AEFIs that were considered as not serious, while 38% reported AEFIs that were classified as serious. Stratifying the results by reference periods, AEFIs were classified as not serious in 53% of reports in period 1% vs. 64% of reports in period 2 (**Table 1**). Overall 25 serious AEFIs’ reports were reported into the RNF (7 in period 1 and 18 in period 2). Among those reports, three cases of autism spectrum disorders (ASDs) were reported. The remaining serious AEFIs’ reports were mainly related to hyperpyrexia, gastrointestinal disorders, persistent crying, and seizure. Thirteen serious AEFIs’ reports (four in period 1 and nine in period 2) were reported very late compared to the date of AEFI’s occurrence (in some cases more than 4 years after AEFI’s occurrence; **Supplementary Table S1**). Among AEFIs’ reports for which outcome was reported (*N* = 55), 78% had a favorable outcome (resolved or improved) and 22% had an unfavorable outcome (resolved with sequelae or unchanged) (**Table 1**).

**Table 2:** AEFIs’ reports associated to individual suspected vaccine.

	Number of Suspected Vaccines in Total AEFIs’ Reports	Number of Suspected Vaccines in AEFIs’ Reports During Period 1 (%)	Number of Suspected Vaccines in AEFIs’ Reports During Period 2 (%)	*p*-Value
Diphtheria, tetanus, pertussis, hepatitis B, poliomyelitis, and *Haemophilus influenzae* b	24	5 (21)	19 (79)	0.806
Measles, mumps, and rubella	24	7 (29)	17 (71)	0.512
Diphtheria, pertussis, and tetanus	12	5 (42)	7 (58)	0.173
Diphtheria, tetanus, pertussis, and inactivated poliomyelitis	8	2 (25)	6 (75)	0.859
Measles, mumps, rubella, and varicella	5	1 (20)	4 (80)	0.888
Varicella virus	2	1 (50)	1 (50)	0.932
Hepatitis B	1	0 (0)	1 (100)	0.587

*The total number of AEFIs’ reports for each vaccine exceeds the total number of AEFIs’ reports (N = 69) because a single report might include more than one suspected vaccine. Database from Campania Region, Southern Italy.*

**FIGURE 2:**
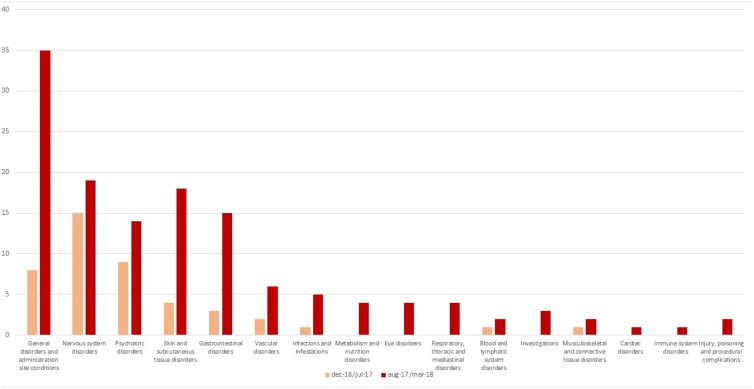
AEFIs distribution by System Organ Class.

Out of 179 AEFIs, more than half referred to the following SOC: “general disorders and administration site conditions” (*n* = 42), “nervous system disorders” (*n* = 30), and “psychiatric disorders” (*n* = 23), although some differences were detected stratifying by reference periods (**Figure 2**). Looking at those SOCs, the most commonly reported PTs were pyrexia, and persistent crying for “general disorders and administration site conditions” (*n* = 31), faint, seizure, and drowsiness for “nervous system disorders” (*n* = 8, each one), and agitation, restlessness, and hallucinations for “psychiatric disorders” (overall, 13 cases) (data not shown). According to the causality assessment, 47% of AEFI/vaccine couples were considered as “consistent causal association to immunization,” 33% as “unclassifiable,” 19% as “non-consistent causal association to immunization,” and 1% was as “indeterminate” (data not shown). Finally, with regard to the source (information available for 67 out of 69 reports), as shown in **Table 1**, 60% of AEFIs’ reports came from healthcare professional while 40% from patient/citizen.

## Discussion

### Overall AEFIs

Along with the rapid increase in the coverage for compulsory vaccines registered in Campania Region after the adoption of the Law 119/2017, there was also an increase in AEFIs’ reporting. According to our results, AEFIs occurred in very young patients. This is not surprising considering that, since newborns and infants have impaired neutrophil, monocytes, and macrophages functions which exposed them to higher vulnerability to pathogens and infectious agents, they are the major target population for multiple vaccines (Simon et al., 2015). In fact, according to the PNPV 2017–2019, vaccines are mainly recommended within the age of six, the school starting age (Piano Nazionale Prevenzione Vaccinale, 2017).

We did not find gender difference. In line with our results, data from an analysis on gender trends in AEFIs’ reporting, using the vaccine surveillance system in Canada, revealed that while gender differences by age group were greatest in adults with a female predominance, no gender differences were observed in children (Harris et al., 2017). On the other hand, a recent review highlighted that infant females seem to be more likely to experience AEFIs to specific vaccines, including hepatitis B, measles, and MMR vaccines. At the same time, other studies revealed that young males have higher rates of immune thrombocytopenic purpura to MMR vaccine and more AEFIs to hepatitis B vaccine than females (Flanagan et al., 2017).

Among AEFIs’ reports for which seriousness degree was indicated, we found that 62% (53% in period 1 and 64% in period 2) were related to not serious AEFIs. These data were encouraging and confirmed by World Health Organization, which stated that vaccines-induced AEFIs are rarely serious (WHO, 2014). Data from further pharmacovigilance studies confirmed our results (Hu et al., 2013; Law et al., 2014). Among serious AEFIs, we found three cases related to ASDs (one associated to MMR vaccine, one to diphtheria, tetanus, pertussis, hepatitis B, poliomyelitis, and *H. influenzae* b vaccine, and one to hepatitis B vaccine). According to the causality assessment, two reports were considered as unclassifiable while for the remaining report the causal association was defined as nonconsistent. During last years, MMR vaccine received the highest attention in this regard (Principi and Esposito, 2016). This association was reported for the first time in 1998 in a study with significant weaknesses (Wakefield et al., 1998). Nowadays, it is largely accepted that there is no association between ASDs and MMR neither with other vaccines (Madsen et al., 2002; WHO, 2003; DeStefano et al., 2004; Smeeth et al., 2004;Mrozek-Budzyn et al., 2010).

We found that the majority of AEFIs reported in Campania region had a favorable outcome. To our knowledge, data on AEFIs outcome in children are very limited. In fact, only the results of the study of Hu et al. (2013) found that out of 792 reports of AEFI related to measles and mumps vaccine all recovered, and there were no reports of death or any sequelae.

In line with our results, data obtained from an analysis of AEFIs reported to the Danish Medicines Agency between 1998 and 2007 revealed that AEFIs most commonly referred to the following SOCs: “general disorders and administration site conditions,” “skin and subcutaneous tissue disorders,” and “nervous system disorders” (Aagaard et al., 2011). Also the results of a further pharmacovigilance study showed that out of 1,742 reports (of which 42% referred to vaccine), ADRs/AEFIs most commonly referred to “general disorders and administration site conditions” and “skin and subcutaneous tissue disorders” (Nogueira Guerra et al., 2015). An analysis of AEFIs’ reports from Switzerland in 2016 showed that, apart from the above mentioned SOCs, AEFIs were also related to SOCs “injury, poisoning, and procedural complications” and “musculoskeletal and connective tissue disorders” (AEFI, 2018). In line with our results, literature data suggested that AEFIs are mainly represented by injection site reactions, febrile seizures, pyrexia, redness, and swelling (Carrasco-Garrido et al., 2004; Swedish Council on Health Technology Assessment, 2009; Aagaard et al., 2011; Donà et al., 2018). Most of those AEFIs can be related to hypersensitivity and anaphylactic reactions, especially when they are associated to dermatological, gastrointestinal, and cardiovascular symptoms (Mahajan et al., 2014; Cheng et al., 2015; Khazaei et al., 2016).

With regard to causality assessment, it is well known that its evaluation is not a simple process, especially for AEFI/vaccine couple and considering the type of event and concomitant factors (Felicetti et al., 2016; Mascolo et al., 2017). Moreover, most of our cases involved multiple vaccinations, which make extremely difficult to attribute causal relationship to one particular vaccine or another.

With regard to the source of reports, we have found that 40% of all AEFI’s reports (33% in period 1 and 43% in period 2) came from patients/citizen. The positive trend for reports from patients is affecting the reporting of AEFIs on the entire Italian territory. In fact, according to data shared by the AIFA, AEFIs’ reports from patient/citizen have increased from 0.3% in 2014 to 2.3% in 2016 (AIFA, 2016, 2018).

### AEFIs Reported Before and After the Law 119/2017

According to our results, there was an increase in AEFIs’ reporting after the adoption of the Law 119/2017 (18 reports in period 1 vs. 51 in period 2).

According to the AIFA, the increase in the number of AEFIs’ reports coincided with the beginning of the parliamentary debate on the decree that preceded the law and the fully activation of Vigifarmaco, a new web reporting and transit platform to the RNF established by AIFA in 2015 (AIFA, 2017). Indeed, in all Italian regions AEFIs’ reports rose from 35% in 2016 to 54% in 2017 and the overall reporting rate for vaccines increased from 7.9/100,000 inhabitants in 2016 to 11.1/100,000 in 2017. This increase should also be put into perspective of the increase in vaccination coverage that has involved the majority of Italian regions, including Campania, which has immediately and firmly adhered to the new obligation. Campania region, in fact, has reached the ministerial fulfillment targets in children aged 0–16, exceeding 95% of vaccination coverage for the hexavalent vaccine, and over 92% for tetravalent and measles vaccines, recovering a historical gap. Therefore, considering the increase in vaccination coverage, the reporting rate for compulsory vaccines was 34.3 reports/100,000 inhabitants and 5.7 reports/100,000 inhabitants for all Italian regions and for Campania, respectively (AIFA, 2017). Consequently, although we observe an increase in AEFIs’ reporting, this should not be viewed as alarming, if we take into account the increase in vaccination coverage and especially positive outcomes deriving from that (improvement of public health, prevention of diseases, protection of frail populations). On the other hand, the increase in AEFIs’ reports could be related to the intense debate that has followed the new law, which was mainly carried out by the so-called Free-Vaxxers group. As previously reported, the rate of ADRs/AEFIs’ reports may increase in response to media attention and increased public awareness, and this is not the first case in Italy (EMA, 2014; Levi et al., 2017; Loharikar et al., 2018). Lastly, in our opinion, the increase in AEFIs’ reports may also be explained by the failure of risk communication campaign on the safety of vaccines. As a matter of fact, since citizens’ decision on immunization must depend on regulatory agencies’, health ministries’, and clinicians’ choices, a huge attention should be paid to their education and information on disease risk and prevention of risk, through an open dialog based on empathy, respect, and transparency (Menditto et al., 2015). In this context, also the management of real world studies (Scavone et al., 2017; Ferrajolo et al., 2018) will help to improve the knowledge on the safety profile of drugs and vaccines.

## Strengths and Limitations

This study has a number of limitations and strengths. First of all, our study is based on spontaneous reporting system, and it is common knowledge that it is affected by boundaries that include under-reporting, inaccurate and incomplete information, and not proper causality attribution, mainly related to lack of clinical data (Ralph Edwards, 1999; Palleria et al., 2013). Considering these intrinsic limitations, we cannot rule out the presence of information that were not listed in AEFIs’ reports and that might have influenced the proper evaluation of each report (i.e., the lack of vaccination’s date or previous/current patient medical conditions which could affect the evaluation of causality assessment). Lastly, since we have decided to perform an analysis of pharmacovigilance data during 16 months of observation in one single Italian region, we have extracted a very limited number of AEFIs’ reports, which should not be considered representative of the other Italian regions, which as reported by the AIFA were characterized by a higher number of AEFIs’ reports.

Despite these limitations, we present a comprehensive evaluation of safety data related to mandatory vaccines in Campania region, using the Italian spontaneous reporting system. While spontaneous reporting system has intrinsic limitation, it is largely accepted that this pharmacovigilance method is a simple and inexpensive tool, that allows to detect rare and serious ADRs/AEFIs not identified during premarketing clinical trials. Furthermore, this method allows to generate safety hypothesis on medicines and vaccines, that shall be confirmed or refuted by *ad hoc* pharmacovigilance studies. Moreover, considering the target population of mandatory vaccines in Italy, we were able to perform a safety analysis on pediatric patients, the most vulnerable population to adverse events’ occurrence. Finally, considering the historic moment that we are living on issues relating to vaccinations, we were able to observe the effects of the Law 119/2017 on AEFIs’ reporting highlighting that in most cases AEFIs were not serious and frequently reported very late compared to their occurrence.

## Conclusion

The Italian Law 119/2017 was adopted with the aim to achieve the highest coverage in immunization, avoiding the return of vaccine-preventable diseases, and to overcome barriers in immunization. Along with the rapid increase in the coverage for compulsory vaccines registered in Campania Region after the adoption of the Law, there was also an increase in AEFIs’ reporting. In our opinion, reasons for this are no doubt numerous, including the increase in vaccination coverage, the huge media attention that has followed the new Law, the increased citizens’ awareness of AEFIs in children as well as a greater empowerment and involvement of patients in pharmacovigilance activities.

Despite those effects on AEFIs’ reporting, there is no doubt about the enormous positive consequences of the Law 119/2017 for public health and especially for frail and vulnerable population, such as patients without a fully working immune system, including those on chemotherapy treatment, patients with HIV, newborn babies, elderly, and hospitalized people. However, a recent Italian amendment of August 2018 overturned the Law on mandatory vaccinations. Thus, when this amendment will become a law, parents will be no longer obliged to have their children vaccinated against severe infectious diseases, such as measles, that in recent years induced serious complications and deaths in Europe, including Italy.

In the context of an effective pharmacovigilance system, the continuous monitoring of vaccine safety and the fully implementation of vaccine-vigilance programs will play a key role in achieving higher confidence in immunization programs and optimal vaccination coverage rate. In order to guarantee the highest safety of immunization programs and to reduce the risk of low coverage, the surveillance of AEFI is a key strategy for regulatory agencies.

## Author Contributions

CS, CR, SB, MB, EM, VO, UT, LS, FR, and AC made substantial contributions to the acquisition, analysis, or interpretation of data for the work; drafted the work and revised it for important intellectual content; wrote the paper; agreed to be accountable for all aspects of the work in ensuring that questions related to the accuracy or integrity of any part of the work are appropriately investigated and resolved; and approved the final version of the manuscript to be published. FR and AC developed the concept.

## Conflict of Interest Statement

The authors declare that the research was conducted in the absence of any commercial or financial relationships that could be construed as a potential conflict of interest.
