# Supplementary material for: Did the New Italian Law on Mandatory Vaccines Affect Adverse Event Following Immunization’s Reporting? A Pharmacovigilance Study in Southern Italy
Source: Front Pharmacol. 2018 Sep 4;9:1003. doi: 10.3389/fphar.2018.01003 (PMC6131571; doi:10.3389/fphar.2018.01003)
Supplement: Supplementary file 1 [file Table_1.DOCX]

Supplementary Material

Article Title

**Cristina Scavone1* and Concetta Rafaniello1*, Simona Brusco1, Michele Bertini1, Enrica Menditto2, Valentina Orlando2, Ugo Trama3, Liberata Sportiello1, Francesco Rossi1, Annalisa Capuano1**

*** Correspondence:** Corresponding Author:

Dr. Cristina Scavone

Campania Regional Centre for Pharmacovigilance and Pharmacoepidemiology - Department of Experimental Medicine – Section of Pharmacology “L. Donatelli”, University of Campania “Luigi Vanvitelli”, Via Costantinopoli 16 – 80138 Naples (Italy)

Phone: 00390815665805

Email: cristina.scavone@unicampania.it

# Supplementary table 1

**Supplementary Table 1. Main features of serious AEFIs**

| **Date of AEFI occurrence** | **Date of entry into RNF** | **Age at vaccination**  **(years or months)** | **AEFI(s)** | **Suspected vaccine 1** | **Suspected vaccine 2** | **Outcome** | **Causality assessment** |
| --- | --- | --- | --- | --- | --- | --- | --- |
| Sept-16 | Dec-16 | 2 years | Lack of awareness, drooling | MMR | - | Unavailable | Non consistent causal association to immunization |
| Mar-17 | Apr-17 | 16 years | Neuropathy | diphtheria, pertussis and tetanus | meningococcal | Improved | Indeterminate |
| May-17 | May-17 | 5 years | Seizure and fall, joint stiffness, agitation | MMRV | diphtheria, tetanus, pertussis and inactivated poliomyelitis | Completely resolved | Consistent causal association to immunization |
| Apr-13 | Jun-17 | 1 year | Language loss, insomnia, pyrexia, Measles, ASD, loss of eye contact | MMR | - | Unavailable | Non consistent causal association to immunization |
| Mar-14 | Jun-17 | 1 year | Antisocial behavior, encephalopathy, stereotypic behavior, compromise psycho-motor development, speech disorder | MMR | - | Unavailable | Non consistent causal association to immunization |
| Dec-15 | Jul-17 | 2 months | Enlarged lymph nodes | MMR | - | Completely resolved | Consistent causal association to immunization |
| Sept-12 | Jul-17 | 10 months | Developmental and psychomotor delays, pyrexia | diphtheria, pertussis and tetanus | pneumococcus | Unavailable | Non consistent causal association to immunization |
| Not available | Sept-17 | not available | Excessive sleep, pyrexia | diphtheria, tetanus, pertussis, hepatitis B, poliomyelitis and H. influenzae b | pneumococcus (13-valent) | Improved | Unclassifiable |
| Dec-06 | Sept-17 | 1 year | Bronchitis, diarrhea, pyrexia, gastroenteritis, IgA deficit, conjunctival hyperemia | MMR | - | Unavailable | Non consistent causal association to immunization |
| Jun-13 | Sept-17 | 6 months | Loss of responsiveness to stimuli, ASD | diphtheria, tetanus, pertussis, hepatitis B, poliomyelitis and H. influenzae b | - | Unavailable | Unclassifiable |
| Mar-17 | Sept-17 | 5 months | Faint, pyrexia | diphtheria, tetanus, pertussis, hepatitis B, poliomyelitis and H. influenzae b | - | Unavailable | Consistent causal association to immunization |
| Oct-15 | Oct-17 | 4 months | Hyperpyrexia | diphtheria, tetanus, pertussis, hepatitis B, poliomyelitis and H. influenzae b | - | Completely resolved | Consistent causal association to immunization |
| Jun-12 | Oct-17 | 1 year | Clotting disorder, diarrhea, ecchymosis, vomiting, petechia, thrombocytopenia | MMR | - | Completely resolved | Consistent causal association to immunization |
| Sept-11 | Dec-17 | 4 months | Weakness, chronic constipation, vomiting, loss of appetite, respiratory tract infection, irritability | diphtheria, tetanus, pertussis, hepatitis B, poliomyelitis and H. influenzae b | - | Unavailable | Unclassifiable |
| Jul-11 | Dec-17 | 2 months | Weakness, constipation, hysteric fits, muscle stiffness, pyrexia, loss of appetite | diphtheria, tetanus, pertussis, hepatitis B, poliomyelitis and H. influenzae b | - | Improved | Unclassifiable |
| Apr-12 | Dec-17 | 11 months | Otitis, laryngospasm, mucositis, disturbed sleep, tonsillitis | diphtheria, tetanus, pertussis, hepatitis B, poliomyelitis and H. influenzae b | - | Unavailable | Unclassifiable |
| Jun-12 | Dec-17 | 1 year | Increased muscle tone, pyrexia | MMR | - | Unavailable | Unclassifiable |
| Nov-17 | Dec-17 | 1 year | Persistent crying, seizure | MMRV | - | Improved | Consistent causal association to immunization |
| Apr-17 | Dec-17 | 3 years | Hyperpyrexia, enlarged lymph nodes, myoclonus, spread urticaria, otitis | MMR | - | Resolved with sequelae | Consistent causal association to immunization |
| Sept-17 | Dec-17 | 12 years | Bradycardia, tonic-clonic seizures, pallor | diphtheria, tetanus, pertussis, hepatitis B, poliomyelitis and H. influenzae b | - | Improved | Consistent causal association to immunization |
| Jan-18 | Jan-18 | 1 year | Rash | diphtheria, tetanus, pertussis, hepatitis B, poliomyelitis and H. influenzae b | pneumococcus (13-valent) | Improved | Consistent causal association to immunization |
| Sept-15 | Feb-18 | 11 months | Delayed motor activity, vacant stare | diphtheria, pertussis and tetanus | pneumococcus | Unchanged | Non consistent causal association to immunization |
| Feb-18 | Feb-18 | 7 years | Redness of the face, hypertonia, nausea, pallor, disturbances of eye movements, head trauma | diphtheria, tetanus, pertussis and inactivated poliomyelitis | MMR | Improved | Unclassifiable |
| Jan-18 | Feb-18 | 1 year | Febrile convulsion, hyperpyrexia | MMRV | - | Completely resolved | Consistent causal association to immunization |
| May-17 | Mar-18 | 1 year | ASD | Hepatitis B | - | Unavailable | Unclassifiable |

ASD: autism spectrum disorder

MMR: measles, mumps and rubella

MMRV: measles, mumps, rubella and varicella
